# The effect of peer-to-peer education on health literacy, knowledge, and adherence to COVID-19 protocols in vulnerable adolescents

**DOI:** 10.1186/s12875-023-01979-w

**Published:** 2023-01-17

**Authors:** Alireza Shoghli, Azam Maleki, Mohammad Reza Masjedi, Mohammadreza Bahrami Hessari, Siavash Khodaei

**Affiliations:** 1 Health Services Management, School of Medicine, Social Determinants of Health Research Center, Zanjan University of Medical Sciences, Zanjan, Iran; 2 Reproductive Health, Social Determinants of Health Research Center, Zanjan University of Medical Sciences, Zanjan, Iran; 3 Pulmonary Diseases, Tobacco Control Research Center (TCRC), Iranian Anti-Tobacco Association, Tehran, Iran; 4 General Psychology, Tobacco Control Research Center (TCRC), Iranian Anti-Tobacco Association, Tehran, Iran; 5 English Language Teaching, administration manager of Iran Non-Communicable Diseases, Tehran, Iran

**Keywords:** Peer influence, COVID-19, Vulnerable populations, Health literacy

## Abstract

**Background:**

The study was done to examine the effectiveness of peer-to-peer education on increasing health literacy, knowledge s, and observance of coronavirus disease (COVID-19) health prevention protocols in vulnerable adolescents.

**Method:**

The study was a one-group intervention (before and after the intervention) that was performed on 1200 vulnerable adolescents living in varamin. The educational intervention was presented to adolescents in a face-to-face session. In the next step, the adolescents were taught the information received by three members of their families. Data were evaluated using a self-designed questionnaire before, and three months after the intervention. The paired t-test was used to compare scores of health literacy, compliance, and knowledge before and after the intervention at a 0.05 confidence level. The Multiple linear regression model was used to determine the predictive factors of observance of COVID-19 preventive behaviors.

**Results:**

The most of adolescents were in the age group of 14 to 18 years (60%) and most of them were girls (61.5%). The most important source of information about COVID-19 disease was radio and television (59.6%). The results showed that the effectiveness of the intervention in increasing the adolescents’ health literacy, knowledge, and adherence to preventive behaviors were 40%, 30%, and 23%, respectively. The effectiveness of the intervention in increasing their families’ health literacy and adherence to the protocols were 11% and 20%, respectively (*p* = 0.001).

**Discussion:**

Involving volunteer adolescents as health ambassadors and transmitting messages and methods of promoting personal protection against COVID-19 epidemics to family members had a significant effect on increasing the knowledge and adherence to the health procedures.

## Background

Since the beginning of December 2019, the COVID-19 epidemic has affected the health and lives of people around the world and has become one of the worst health and economic crises of contemporary history. There is limited medication for the treatment of COVID-19 [1]. One of the practical measures to increase resilience and guide society to return to normal activities is to help strengthen the observance of personal protective behaviors among different groups of society, especially vulnerable groups and people who cannot observe the physical distance due to their work and life conditions [2]. Marginalized (suburbanite) populations, foreign people, and refugees are more vulnerable during the COVID-19 epidemic for many reasons [3]. Iran hosts a large number of refugees, mostly from Afghanistan and Iraq. Also, according to an estimate, up to 2 million Afghans do not have legal documents to stay in Iran [4]. Most immigrants reside in inner-city settings [5]. The Iranian government implements many actions to improve the health of refugees in the country, mainly using domestic resources. Recently, in response to the health needs of immigrants, new policies including social insurance and improving the access and quality of medical services for immigrants have been started in Iran [6].

Refugees may be at risk of discrimination and stigma by local people as potential carriers of the coronavirus [7]. In addition, due to quarantine restrictions, access to health, mental, and social services, and communication and language barriers, access to information related to COVID-19 is restricted in these individuals. Thus, refugees’ right to know about COVID-19 prevention and protection methods is weakened [8–10].

Health literacy is the main factor for following disease prevention behaviors that show the ability of persons to access, understand, and use health-related information to make decisions in health-related fields [11]. It is a socio-cognitive skill, that enables individuals to be well aware of the risks, resources, and health advice associated with diseases and, ideally, to act according to principles that lead to the promotion of public health [12]. In addition to increasing knowledge and awareness of the disease and preventative behaviors, COVID-19 health literacy facilitates the recognition of correct information from incorrect ones and allows individuals to make informed health decisions and protective behaviors during epidemics [11]. In a study, Australian adults with poor health literacy about COVID-19 had less self-efficacy in adopting infection prevention behaviors [13].

Another study was investigated in 2020 to examine the health literacy of marginalized people in the COVID-19 widespread, the most urgent health and social problems of these families during the COVID-19 epidemic included feelings of food uncertainty, economic issues, and limited access to health-center and COVID-19 testing centers. Due to the ethnic diversity in the population group of the mentioned study, education in different languages and dialects in the target communities was suggested to provide better services [14].

Health literacy is usually affected by people’s socioeconomic and employment status. People living below the poverty line have lower health literacy comparing other social classes [15]. The general public and governments often pay high costs to compensate for inadequate health literacy in the community. Health literacy is not just an individual responsibility. The health system and related sectors must provide the necessary facilities to promote health literacy on a larger scale and for a larger segment of the community [16].

Planning is very important to change the attitudes and behaviors of people in the community [17]. Furthermore, the experience of the fifth wave of the COVID-19 epidemic in Iran shows that the rate of compliance with the health protocols in the community is not adequate, and to increase compliance with the protocols in different groups, it is necessary to use different approaches and educational methods [18, 19].

Previous data recommends that the use of "peer-to-peer models" to educate vulnerable populations has been successful in some health areas, such as the prevention of infectious diseases [18, 20, 21], "physical activity", psychological health, feeding, sexual disease, smoking, alcohol use, and illegal drugs [22]. Peer support, and the integration of peer relationships into health care delivery, is a concept that is important to scientists and physicians today as it shifts the focus from disease treatment to health promotion. Peer education is a model of health education in which people share their information, opinions, or skills for various reasons, including similarity in age, sex, living environment and experience, and even cultural and social situations [23]. Using the peer-to-peer approach provides unique opportunities to increase empathy, strengthen social capital, and create a sense of equality [24]. Due to the benefits of peer training in creating effective communication, empathy, and a sense of relaxation, it has been registered by the World Health Organization as an effective method for changing people's behavior. To increase personal health and empower people for self-care, Iran's Ministry of Health has started training programs for health ambassadors since 2013. The self-care program is mostly related to heart attack disease, stroke, and cancer, healthy lifestyle, and self-care week by week during pregnancy. This group is not only responsible for themselves, but also for their family and society, and by following a healthy lifestyle, they increase the positive impact of society from various pieces of training and advertisements in the field of spreading health behaviors [25]. Meanwhile, still, there is no adequate information on the effectiveness of educating vulnerable families by adolescents to increase compliance with COVID-19 prevention behaviors. Accordingly, in the study, the peer-to-peer education model was used to empower adolescents and their families.

### The purpose of the study

The study was done to determine the effectiveness of the "peer-to-peer model" in increasing health literacy, knowledge, and adherence to health protocols for COVID-19 in vulnerable adolescents.

## Methods

### Setting and participants

The study was done in the form of a one-group intervention study (before and after the intervention). This study was based on a project approved by Zanjan University of medical sciences, 2020–2021.

The statistical population consisted of three vulnerable adolescent groups (prisoners’ families, foreign nationals, and poor families) in Varamin and suburbs that were selected using purposive sampling. For better access to the target population included (prisoners’ families, foreign nationals, and poor families), we provided a list of families who are supported by public organizations such as the Relief Committee, the State Welfare Organization, and other supporting institutions. Participants were invited by telephone to participate in the study. If they do not have a family, live in the place, and live with a group of foreign nationals or refugees, they were also invited to participate in the study.

Taking into account the desired adherence to health protocols of COVID-19 rate at the level of 50%, ɑ = 0.05(the first type error), and d = 0.05(the accepted error), the sample size was estimated as 384 people, which with a prediction of 10% attrition, increased to 400 people in each population group. In this study, 400 trained adolescents were recruited as health ambassadors to deliver health messages related to COVID-19 to families. In this way, each adolescent taught what he/she had learned to at least three persons (their family members or peers). Therefore, the total sample size consisted of 1200 people.

Inclusion criteria for adolescents were as follows: age ≥ 8 years, literacy, voluntary participation, commitment to attend the course with the approval of the caretaker or legal guardian, membership in the families of foreign nationals, under the auspices of relief or welfare organizations or other support institutions, having appropriate communication skills and the ability to convey concepts to others and having a contact number (phone). Each participant was excluded from the study if he/she did not attend the briefing session, disconnected for more than one month, and did not wish to continue the cooperation.

### Procedure/intervention

**Fig. 1:**
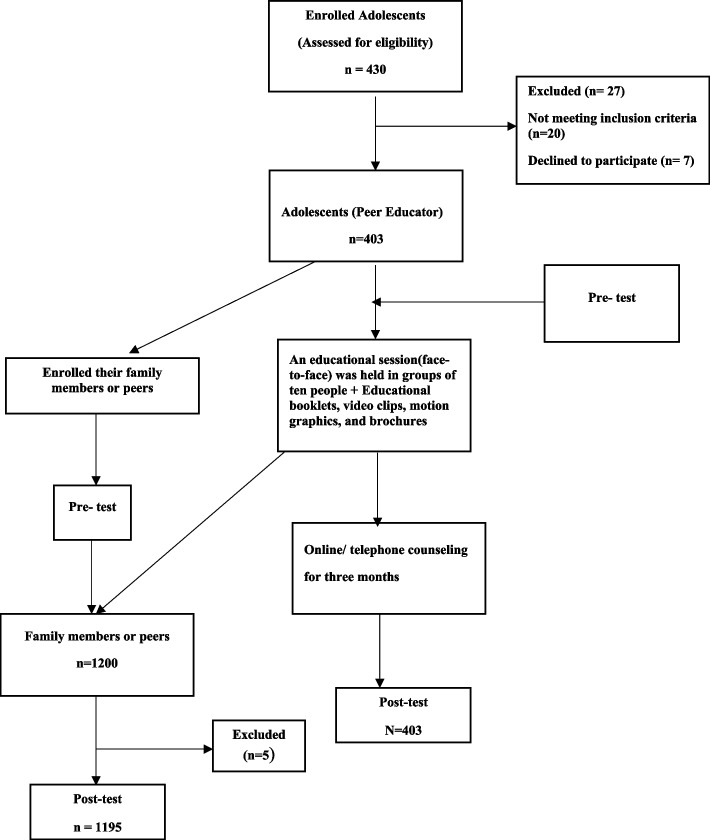
Procedure and Intervention

The educational content was compiled according to the latest instructions of the World Health Organization (WHO) and the Ministry of Health and was provided to the participants in the form of educational booklets, video clips, motion graphics, and brochures. These topics included an introduction to COVID-19, the nature of the coronavirus and how it spreads and induces disease, signs, and symptoms of COVID-19, ways to prevent transmission of infection, a review of health guidelines and protocols, how to access information resources related to the COVID-19 epidemic, how to read, understand, and evaluate information obtained from various sources related to COVID-19, preventing misconceptions and avoiding irrational behaviors related to COVID-19, and strengthening communication skills and knowledge transfer to other people. The content was prepared in the local dialect and Persian language (Fig. 1).

### Peer educator

The adolescents were taught the provided content in a face-to-face session in groups of ten people according to the COVID-19 prevention protocols. The meeting place was well-ventilated, all participants wore masks, and the chairs were arranged at proper physical distance to prevent possible transmission of the virus. A special educational booklet for health ambassadors was also provided to participants at the beginning of the session. The adolescents were then asked to pass on what they had learned about the concept of peer education, and coping with COVID-19 to at least three other persons (their family members or peers) under the supervision of the research team. Throughout the program, all health ambassadors received online counseling (need-line). The performance of the health ambassadors was monitored via telephone by the advisory team until the end of the course. During the follow-up period, protective equipment and disinfectants were prepared and distributed to the target populations.

### Data collection

Data were evaluated using a self-designed questionnaire before, and three months after the intervention. The questionnaires were completed in two methods face-to-face (pen and paper) for the adolescents and the phone interview for their families by a person not involved in the research process.

### Demographic questionnaire

The questionnaire included age, sex, level of education, history of COVID-19 in adolescents and history of other diseases in their family members, family size, and channels of gathering information about COVID-19.

### Questionnaire of adherence to preventive behaviors, health literacy, and knowledge of COVID-19 health protocols

The questionnaire was designed in two versions for adolescents and their families. The adherence to preventive behaviors questions included 10 questions for adolescents and 11 questions for families such as "Clean and clean contaminated surfaces, Eating Outdoors—Transportation—Unnecessary Outdoor Presence—Going to the Parties—Making Proper Ventilation at Home – Wearing Masks—Using Gloves in Contact with Contaminated Surfaces—Hand Washing-—disinfect food before use—Social distance at least one meter or avoid close contact with people". The questions were scored on a four-point Likert scale from 0 to 3.

The health literacy questions included 6 questions for adolescents and 10 questions for families. The questions were scored on a five-point Likert scale from 0 to 4. Therefore, the minimum and maximum scores ranged from 0 to 24 for adolescents and 0- 40 for their families.

Examples of health literacy questions: "I am comfortable reading about COVID-19 in printed sources such as newspapers or magazines", "I'm only comfortable getting information about COVID-19 disease on TV", "I know where I can find reliable information about COVID-19 disease", "I know where I can get personal protective equipment to prevent COVID-19 disease", "I easily get health advice from health workers, friends or TV", "I can easily recognize messages from newspapers, friends, magazines or TV is true", "I follow my doctor's instructions about COVID-19 disease"," I behave in such a way as to prevent others from catching the coronavirus", and" I see a doctor or health care provider as soon as I see symptoms of COVID-19 disease". The questions were scored on a five-point Likert scale from 0 (never) to 4 (always). Finally, knowledge questions included 5 questions for adolescents such as "I know when we have symptoms of COVID-19 disease, we should see a doctor or health care provider as soon as", "I know hand washing, using a disinfectant, using masks, observance, distance from others, and quarantine is the most important way to prevent COVID-19 disease", "I know that asymptomatic people also transmit the coronavirus to others for up to two weeks", " I know close contact with people with COVID-19 disease is dangerous and should be avoided"," I know the most common symptoms of COVID-19 disease are cough, sore throat, fever, shortness of breath".

In the study, the content validity ratio (CVR) was calculated for each item on the opinions of 5 experts. Seven of the items had a full score and the rest of them had a score of 0.94. The average CVR score was 0.989, which was reported to be 0.99 after rounding. The content validity ratio was compared with the existing criterion via the Lavsheh table. The number of the Lavsheh table is 0.99 for 5 people and the obtained number indicates that it is necessary to have a relevant question with an acceptable level of statistical significance in the tool. To calculate the content validity index (CVI) of each item, the questions were scored separately on a four-point Likert scale in terms of three criteria of simplicity, relevance, and clarity. In the present study, the CVI scores for each question were higher than 0.79, indicating a satisfactory condition. In formal validity, the impact score was more than 1.5. Therefore, all questions were identified as appropriate, and thus, all were retained.

In this study, the total Cronbach’s alpha coefficient of the instrument was 0.70. The intraclass correlation coefficient of all items was 0.91 for a two-week interval.

### Statistical methods

SPSS software version 16 was used for data analysis. Descriptive statistics indices were used to describe the information. Data were normally distributed based on the Kolmogorov–Smirnov test. The paired t-test was used to compare scores of health literacy, compliance, and knowledge before and after the intervention at a 0.05 confidence level. The Multiple linear regression model was used to determine the predictive factors of observance of COVID-19 preventive behaviors. In the multiple linear regression model with the Inter method at a 95% confidence interval, the variables of age, sex, level of education, population group were considered as predictors factors of observance of COVID-19 preventive behaviors.

## Results

### Baseline data

Demographic characteristics of the adolescents participating as health ambassadors were as follows. According to the results presented in Table 1, most of the adolescents were in the 14 to 18 years age group (60%), women (61.5%), sixth grade and lower grades (elementary school) (45.2%), from families of four (35.2%), urban resident (60.5%), and poor families (49.6%) (Table 1).

**Table 1:** Demographic characteristics of the adolescents by age, sex, grade, family size, place of residence, and population group (*n* = 403)

Variable		Frequency	Percentage
Age (year)	8–14	242	60
	15–19	129	32
	20 and over	32	8
Sex	female	246	61.5
	male	154	38.5
Grade	6th and lower	173	45.2
	7th–9th	118	30.8
	10th–12th	92	24.0
Family size	3 and lower	77	19.1
	4	142	35.2
	5	108	26.8
	6	49	12.2
	7	12	3.0
	8 and higher	15	3.7
Place of residence	urban	244	60.5
	rural	156	38.7
Population group	prisoners’ families	100	24.8
	foreigners	103	25.6
	poor families	200	49.6

Demographic characteristics of the participants as peers or family members were as follows. According to the results presented in Table 2, the highest percentage of the participants as peers or family members were up to 19 years (26.6%), women (62.8%), with an associate degree (26.9%), from families of four (39.1%), urban resident (64.1%), and poor families (49.6%). Hypertension was the most common disease reported by the participants (Table 2).

**Table 2:** Demographic characteristics of the adolescents' family members or peers by age, sex, education level, family size, place of residence, and population group (*n* = 1195)

Variable		Frequency	Percentage
Age (year)	9 and below	49	4.1
	10–19	318	26.6
	20–29	171	14.3
	30–39	276	23.1
	40–49	235	19.7
	50–59	87	7.3
	60 and over	59	4.9
Sex	female	750	62.8
	male	445	37.2
Education level	illiterate	41	3.4
	primary school	125	10.5
	middle school	169	14.1
	high school	219	18.3
	associate degree	321	26.9
	bachelor	34	2.8
	postgraduate	5	0.4
	other (children)	39	3.3
Family size	3 and lower	224	18.7
	4	467	39.1
	5	284	23.8
	6	148	12.4
	7	33	2.8
	8 and higher	39	3.3
Place of residence	urban	766	64.1
	rural	429	35.9
Population group	prisoners’ families	300	25.1
	foreigners	302	25.3
	poor families	593	49.6
Suffering from diseases	diabetes	35	2.9
	hypertension	48	4.0
	heart diseases	25	2.1
	respiratory diseases	12	1.0
	malignancies	5	0.4
	other	25	2.1

### Sources of information gathering about COVID-19

**Fig. 2:**
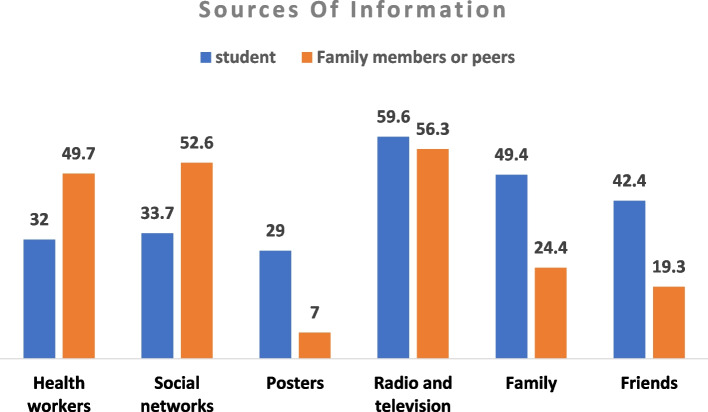
Sources of information about COVID-19 disease

The sources of information in the group of adolescents included radio and television (59.6%), family members (49.4%), friends and relatives (42.4%), social networks (33.7%), health workers (32%), and posters and announcements in the city (29%), respectively. Also, the most sources of data about COVID-19 in the group of peers and family members included radio and television (56.3%), social networks (52.6%), health workers (49.7%), and family (24.4%), respectively (Fig. 2).

The results showed that 91.6% of the contributors were satisfied with the method the training course was held. Also, 86.8% of these people believed that participation in the training course had a role in increasing compliance with the protocols. Moreover, 86.4% of them believed that the distribution of protective equipment was effective in this regard.

### Comparison of health literacy, knowledge, and observance of COVID-19 preventive behaviors (before, after)

Data were normally distributed based on the Kolmogorov–Smirnov test. The results showed that the mean score of health literacy increased from 13.23 before the intervention to 18.62 after it, and therefore, the effectiveness of the intervention in increasing health literacy was 40%. Also, the mean score of knowledge before the intervention was 12.24, which increased to 15.95 after it. Accordingly, the effectiveness of the intervention in raising knowledge was 30%. The mean score of adherence to COVID-19 prevention behaviors before the intervention was 14.95, which increased to 18.35 after it. Hence, the effectiveness of the intervention in increasing compliance with mentioned behaviors was 23% (Table 3).

**Table 3:** Comparison of the means of health literacy, knowledge level, and adherence to protocols before and after the intervention (*n* = 403)

Variables	Intervention	Mean ± SD	Mean Difference	T	*P*-Value*
health literacy	before	23.13 ± 73.7	6.53	6.41	0.001
	after	18.62 ± 4.97			
knowledge level	before	12.24 ± 6.91	3.71	14.49	0.001
	after	15.95 ± 4.39			
adherence to protocols	before	14.95 ± 9.12	3.41	10.07	0.001
	after	18.35 ± 9.78			

^*^The *p*-value is reported based on the results of the paired sample t-test

Comparison of the mean scores of health literacy, knowledge, and adherence to COVID-19 preventive behaviors in the peers or family members, before and after the intervention.

Data were normally distributed based on the Kolmogorov–Smirnov test. The results showed that the mean score of health literacy in families increased from 28.81 before the intervention to 32.15 after it, and as a result, the effectiveness of the intervention in increasing health literacy was 11%. The mean score of observance of COVID-19 preventive behaviors was 19.24 before the intervention, which increased to 23.26 after it. Accordingly, the effectiveness of the intervention in increasing compliance with these behaviors was 20% (Table 4).

**Table 4:** Comparison of the means of health literacy and adherence to protocols among the family members before and after the intervention (*n* = 1195)

Variables	Intervention	Mean ± SD	Mean Difference	t	*p*-value*
Health Literacy	Before	28.81 ± 6.35	3.34	-37.04	0.001
	After	6.71 ± 32.15			
Adherence to Protocols	Before	19.24 ± 4.70	4.02	-34.68	0.001
	After	23.26 ± 6.84			

^*^The *p*-value is reported based on the results of the paired sample t-test

### The predictive factors of observance of COVID-19

The Multiple linear regression model showed population group, education, and sex were the predictive factors of observance of COVID-19 preventive behaviors. The observance of COVID-19 protocols in the poor family group was 0.18 times more than in the next two groups. While the male gender and grade 10–12 were -0.15 and -0.13 times lower than the rest (Table 5).

**Table 5:** The predictive factors of adherence to protocols among adolescents

Model	Unstandardized Coefficients		Standardized Coefficients	t	*P* value	95.0% Confidence Interval for B	
	B	Std. Error	Beta			Lower Bound	Upper Bound
Population group	1.38	0.38	0.18	3.58	0.001	0.62	2.13
Education	-1.03	0.51	-0.13	-1.99	0.047	-2.05	-0.01
Sex	-1.96	0.64	-0.15	-3.04	0.002	-3.22	-0.69
Age	1.02	0.64	0.10	1.59	.111	-.23	2.29

the multiple linear regression model

### The rate of COVID-19 cases

Comparing the rate of COVID-19 infected cases before and after the intervention showed that the number of cases among adolescents decreased from 57 to 55 cases. Of this amount, 51 people were treated at home and 4 people at the hospital.

## Discussion

Vulnerable and marginalized populations are among the groups most at risk for COVID-19 disease. Because of their living and working conditions, they may be less likely to comply with quarantine conditions. Hygiene and self-care are among the most essential ways to control the disease in most communities, particularly low-income and vulnerable groups. The results of the study showed that attracting volunteer adolescents as health ambassadors to convey messages and methods of personal protection against COVID-19 to family members, along with providing situation-based counseling, is very effective in increasing knowledge, health literacy, and adherence to health protocols in adolescents and their families.

Furthermore, this research shows that adolescents, as one of the effective channels for informing about COVID-19, can play an effective role in improving their health and that of their families. Adolescents are familiar with the language and culture of their families, know their needs well, and live with them for a long time. Acquaintance with COVID-19 self-care guidelines helps them pass that knowledge on to their families and friends in addition to taking care of themselves. Also, institutionalizing health behaviors during adolescence leads to better performance in adulthood.

The results showed that the effectiveness of the intervention in increasing the adolescents’ health literacy, knowledge s, and adherence to health protocols were 40%, 30%, and 23%, respectively. Meanwhile, the effectiveness of the intervention in increasing the health literacy of families and their adherence to health protocols were 11% and 20%, respectively. The higher impact of the intervention on the adolescents compared to their families may be due to the adolescents’ limited communication skills or their restricted abilities to convey messages to their families. Also, a significant percentage of the family members who participated in the study were illiterate, which in itself can contribute to their low health literacy.

The results of the present study are consistent with the results of a study by Wieland et al. in 2020. In their study, they showed that training people with their peers to understand the risk of COVID-19 disease and increase the adaptation of vulnerable groups increased the understanding of the risks for themselves and the community, and thus increased self-care [26]. Kaim et al. demonstrated that educational interventions, such as the brief video were an effective method for educating people in the community [27].

Ghaffari et al. showed that 54.9% of the suburban population had adequate health literacy. Health literacy was higher in men compared to women, but this difference was not statistically significant. Also, self-care was at a moderate level in 57.4% of the participants and a low level in the rest. In addition, there was a significant relationship between health literacy and happiness, social functioning, and self-care [28].

In another study, Shakiba et al. examined the effect of peer-to-peer self-care training on the number of visits to the physician for the treatment of minor illnesses. They showed that the number of visits to the physician in the experimental group was significantly reduced after the intervention. Also, a significant difference was reported in the level of knowledge in the experimental group before and after the intervention [29]. In 2021, Pasek et al. evaluated the effect of peer-to-peer education on the acceptance of COVID-19 vaccination among adolescents. In this study, a training session was held for 13 medical adolescents and 51 non-medical adolescents through health ambassadors. The results of the study showed that the willingness to accept the COVID-19 vaccine in adolescents increased from 31.8 to 35.2 [30]. The results of the above study are consistent with the results of the present study and indicate the positive effects of using the peer-to-peer education model in changing behaviors and improving health-related outcomes. These results can be important for health planners. Although the health ambassadors' program in Iran is being implemented in the health system as the “National Self-Care Program”, it is not implemented accurately or does not meet the required standards for various reasons, such as the lack of knowledge or communication skills health ambassadors and the unwillingness of people in some regions to cooperate with the program. As a result, it is not possible to truly evaluate the personal self-care program—the core of which is the training of families’ health ambassadors and providing deep education for them [29].

Zareipour et al. found that only 25.5% of the health ambassadors who participated in their study had adequate health literacy. Also, health literacy rates were significantly higher in men, people under 35, employees, and people with a university education. There was a significant positive correlation between health literacy and the self-efficacy of health ambassadors [25]. These findings indicate that to improve the effectiveness of peer education, in addition to the interest of individuals to play the role of health ambassador, empowerment courses should be held to increase health literacy and strengthen the communication skills of people involved in this process.

Evidence shows that the use of peer-to-peer models to educate vulnerable populations has been successful, especially in some health aspects such as the prevention of communicable or non-communicable diseases [31, 32]. Moreover, the peer education model has been highly effective in caring for patients rescued from COVID-19 [33]. Applying the peer education approach provides unique opportunities to increase empathy, strengthen social capital, and create a sense of equality [24]. Another potential of health ambassadors, in addition to the role of self-care or monitoring compliance with COVID-19 health protocols in the family, workplace, or community, is to increase health literacy in various social groups [25].

According to the findings of the study, the most important sources of information gathering about COVID-19 in the group of adolescents included radio and television (59.6%), family members (49.4%), friends and relatives (42.4%), social networks (33.7%), health workers (32%), and posters and announcements in the city (29%), respectively. Also, the most important sources of information gathering about COVID-19 in the group of peers and family members included radio and television (56.3%), social networks (52.6%), health workers (49.7%), and family (24.4%), respectively. People have a lot of confidence in the information they receive from credible sources, and these resources are appropriate communication channels for providing information that will increase people’s health literacy. The results of the study show that national radio and television have been the most important communication channels for receiving information. Since the outbreak of COVID-19, the Ministry of Health and Medical Education in Iran has been the main source of information-dissemination on COVID-19 for all people and all groups of the community. But the quality of these media and their relevance to people with low health literacy is also very significant.

According to evidence published on the website of the Ministry of Health and Medical Education regarding the quality of educational media related to COVID-19, the effectiveness of such information dissemination was not as high as expected. According to the criteria for designing educational media for people with low health literacy, it can be said that the performance of these media has been inadequate and below the acceptable level, especially regarding observing the points related to the main message, calling for action, and language. In contrast, issues related to information design and behavioral recommendations were significantly higher than expected [34]. Due to the importance of the subject, the evaluation of educational content of other media, especially social networks, can also be considered by health officials and planners.

In the present project, in addition to increasing knowledge and health literacy, other motivational sources were used to increase compliance with the health protocols. In this regard, 91.6% of the participants were satisfied with the way the training course was held and 86.8% believed that participation in the course had a role in increasing compliance with the protocols. Also, 86.4% of them believed that the distribution of protective equipment was effective in this regard. Given that the target population of the study was disadvantaged strata, easy and low-cost access to protective equipment could play a role in increasing compliance with health protocols.

### Strengths of study

Among the strengths of this study, one could point to the large sample size, the use of a standard questionnaire, and the variety of samples from different vulnerable groups, such as foreign nationals living in Varamin city. Also, in the study, a combination of educational methods with maximum tractability was used for education. The educational content was prepared or updated following the latest authoritative scientific sources—including guidelines published by the World Health Organization—in the form of booklets, brochures, educational clips, and motion graphics. In addition, the educational content was organized in the form of local and foreign dialects and languages so that local people and foreigners could also benefit from it.

### Limitations

The data collection tool was self-report, and to increase the accuracy of the data, this limitation is expected to be controlled by explaining the aims of the study and the importance of accuracy in answering questions. The selection of individuals in the present study was not random and due to ethical considerations and to avoid the exclusion of certain groups from the training process, the study was designed in the form of a one-group intervention (before and after the intervention). Therefore, the generalization of the results of this study should be done according to the mentioned limitations.

The reluctance of vulnerable groups to participate in this study was another limitation for researchers. To overcome this limitation, an attempt was made to explain the purposes of the study and the role of individuals in maintaining the health of the family and the community to the audience to increase the likelihood of their participation. At the time of implementation of this study, there is insufficient data about the Covid-19 vaccine. Also, Iran's government along with the homegrown vaccines announced that permission for Sputnik V vaccines was issued to import to Iran. Vaccination of first-line healthcare workers in Iran began in February 2021 with a limited number of Russian Sputnik V vaccines. Therefore, the performance of the participants regarding the Covid-19 vaccine has not been investigated.

In the present study, the economic and social effects of COVID-19 disease have not been investigated. Consequently, it is suggested that in future studies, the effect of those factors on the degree of compliance with health protocols should be considered.

## Conclusions

Involving volunteer adolescents as health ambassadors and conveying messages and methods of personal protection against the COVID-19 pandemic to family members was very effective in increasing knowledge of adolescents and families and their adherence to the health protocols and therefore, it seems an acceptable approach. Interventions with different approaches are recommended for the elderly and large families with low education levels.

## Data Availability

The dataset used in the present study is available from the corresponding author upon reasonable request.

## Abbreviations

COVID-19: Coronavirus disease
CVR: Content validity ratio
CVI: Content validity index
SGP: Small grants program
UNDP: United nations development program
IATA: Iranian Anti-Tobacco Association
